# Shared Genetic Liability and Causal Associations Between Major Depressive Disorder and Cardiovascular Diseases

**DOI:** 10.3389/fcvm.2021.735136

**Published:** 2021-11-11

**Authors:** Fuquan Zhang, Hongbao Cao, Ancha Baranova

**Affiliations:** ^1^Wuxi Mental Health Center of Nanjing Medical University, Wuxi, China; ^2^Department of Psychiatry, The Affiliated Brain Hospital of Nanjing Medical University, Nanjing, China; ^3^Institute of Neuropsychiatry, The Affiliated Brain Hospital of Nanjing Medical University, Nanjing, China; ^4^School of Systems Biology, George Mason University, Fairfax, VA, United States; ^5^Research Centre for Medical Genetics, Moscow, Russia

**Keywords:** major depressive disorder, cardiovascular disease, Mendelian randomization, polygenic overlap, stroke

## Abstract

Major depressive disorder (MDD) is phenotypically associated with cardiovascular diseases (CVD). We aim to investigate mechanisms underlying relationships between MDD and CVD in the context of shared genetic variations. Polygenic overlap analysis was used to test genetic correlation and to analyze shared genetic variations between MDD and seven cardiovascular outcomes (coronary artery disease (CAD), heart failure, atrial fibrillation, stroke, systolic blood pressure, diastolic blood pressure, and pulse pressure measurement). Mendelian randomization analysis was used to uncover causal relationships between MDD and cardiovascular traits. By cross-trait meta-analysis, we identified a set of genomic loci shared between the traits of MDD and stroke. Putative causal genes for MDD and stroke were prioritized by fine-mapping of transcriptome-wide associations. Polygenic overlap analysis pointed toward substantial genetic variation overlap between MDD and CVD. Mendelian randomization analysis indicated that genetic liability to MDD has a causal effect on CAD and stroke. Comparison of genome-wide genes shared by MDD and CVD suggests 20q12 as a pleiotropic region conferring risk for both MDD and CVD. Cross-trait meta-analyses and fine-mapping of transcriptome-wide association signals identified novel risk genes for MDD and stroke, including *RPL31P12, BORSC7, PNPT11*, and *PGF*. Many genetic variations associated with MDD and CVD outcomes are shared, thus, pointing that genetic liability to MDD may also confer risk for stroke and CAD. Presented results shed light on mechanistic connections between MDD and CVD phenotypes.

## Introduction

Collectively, mental disorders and cardiovascular diseases (CVD) account for a large proportion of the total disability and morbidity worldwide (1, 2). Major depressive disorder (MDD), commonly referred to as depression, is characterized by the persistence of low mood. MDD is the most prevalent mental disorder and is accompanied by considerable morbidity, mortality, and a high risk of suicide (3). At some point during the lifetime, it affects 1 out of 5 adults (4). Major forms of CVD include hypertension, coronary heart disease, heart failure, stroke, and atrial fibrillation. High-rate of co-morbidity between depression and CVD is well-acknowledged; patients with depression are more likely to develop CVD, and patients with CVD have higher depression scores than the general population (5). Among patients with CVD, depression is a major contributor to increased healthcare cost, mortality, and reduced quality of life (6, 7), and is also considered an independent risk factor for major adverse cardiovascular events (8). Specifically in coronary heart disease patients, the prevalence of depression is reported at 15–23% (9).

A prevailing measure of quantifying the genetic relationship between two traits is a genetic correlation coefficient, with its sign indicating the direction of the shared genetic effect. However, when dealing with mixtures of effect directions across shared genetic variants, the genetic correlation analyses may be underpowered (10). Polygenic overlaps were recently proposed to measure the fraction of genetic variants causally associated with both traits over the total number of causal variants across a pair of traits involved (10).

In previous studies, MDD has been reported to be genetically correlated with coronary artery disease (CAD) (11). Nevertheless, whether these associations are causal remains to be seen. Mendelian randomization (MR) approach tests for causative association between an exposure and an outcome by utilizing genetic variants as instrumental variables (12, 13). Several frameworks have been proposed for MR analysis, including MR-Egger methods (14). Recently, a powerful GSMR (Generalized Summary-data-based Mendelian Randomization) suit was developed to account for linkage disequilibrium (LD) by leveraging power from multiple genetic variants (15). The GSMR framework is increasingly employed in recent analyses (16–20), with reports of the causal effects of MDD on small vessel stroke, ischemic heart disease, and CAD already available (21–23).

In this study, we evaluated genetic correlation and polygenic overlap between MDD and eight cardiovascular conditions and reported their causal associations. To achieve this, a multi-SNP MR analysis was run on summary GWAS datasets. Across MDD and CVD, pleiotropic genes were extracted by comparing genome-wide genes reported for each trait. Then, in cross-trait meta-analyses, pleiotropic genomic loci shared between MDD and stroke were identified, followed by prioritizing putative risk genes by leveraging a multi-tissue eQTL database.

## Method

### GWAS Summary Datasets and Quality Control

The summary results of GWAS of MDD (20) and seven cardiovascular conditions—CAD (24), heart failure (25), atrial fibrillation (26), stroke (27), systolic blood pressure (28), diastolic blood pressure (28), and pulse pressure measurement (28)—were used for the analyses. The summary result of GWAS of CVD (29) was used in the validation stage. The CVD dataset included a mixture of multiple cardiovascular diseases recruited by the UKB (29). Participants from these datasets were either of European origins (for traits of MDD, stroke, heart failure, CVD, and blood pressure) or mainly of European origins (for atrial fibrillation and CAD). Condition-specific sample sizes have ranged from 332,477 to 977,323. Each SNP was analyzed across pairs of datasets after exclusion of all SNPs with conflicting alleles, and effect harmonization. Detailed information on the datasets included in this study is summarized in Table 1 and Supplementary File.

**Table 1:** Summary information of the datasets.

**Trait**	**Author**	**Year**	**PMID**	**Cases**	**Controls**	***N***
Major depressive disorder	Wray et al.	2018	29700475	135,458	344,901	480,359
Coronary artery disease	Nelson et al.	2017	28714975	71,602	260,875	332,477
Heart failure	Shah et al.	2020	31919418	47,309	930,014	977,323
Atrial fibrillation	Roselli et al.	2018	29892015	65,446	522,744	588,190
Stroke	Malik et al.	2018	29531354	40,585	406,111	446,696
Systolic blood pressure	Evangelou et al.	2018	30224653	NA	NA	745,820
Diastolic blood pressure	Evangelou et al.	2018	30224653	NA	NA	757,601
Pulse pressure	Evangelou et al.	2018	30224653	NA	NA	745,820
Cardiovascular disease	Sudlow et al.	2015	25826379	14,510	97,828	112,338

### Genetic Correlation and Polygenic Overlap Analysis

GWAS summary results were utilized to extract the genetic correlation of MDD with cardiovascular conditions using LD score regression software (LDSC, v1.0.1) (30, 31). Polygenic overlaps were analyzed by MiXeR v1.2 using default parameters (10). The MiXeR pipeline evaluates the number of shared and trait-specific causal variants between two traits, while accounting for effects of LD structure, minor allele frequency (MAF), sample size, cryptic relationships, and sample overlap. The total number of causal variants was 22.6% of the total estimate, which accounts for 90% of SNP heritability for each trait.

### MR Analyses

We examined causal effects between MDD and the seven cardiovascular conditions, namely, CAD, heart failure, atrial fibrillation, stroke, systolic blood pressure, diastolic blood pressure, and pulse pressure measurement. GSMR v1.0.9 was used to infer bidirectional causal associations between MDD and the cardiovascular conditions, with causal effects of cardiovascular conditions on MDD being called reverse Mendelian randomization (15). Instrumental variants were selected based on default *P* ≤ 5 × 10^−8^. When the threshold was surpassed by <10 SNPs, a *P*-value threshold of 1 × 10^−5^ was used. As pleiotropy is known to serve as a potential source of bias and, therefore, an inflated estimation in an MR analysis (32), we used the HEIDI-outlier approach, which detects and eliminates genetic instruments with apparent pleiotropic effects on both the risk factors and the disease (15, 33). Multiple tests were corrected by FDR, with significant causal association detected at FDR < 0.05. A detailed description of the MR is provided in the Supplementary Methods section.

### Comparison of Genome-Wide Genes Shared Between MDD and CVD

GWAS results were obtained for MDD and four types of CVD from the GWAS Catalog database (34). For stroke, we combined the following labels: stroke as such, large artery stroke, small vessel stroke, cardioembolic stroke, and ischemic stroke. Analysis of gene overlaps among the five traits was conducted using the R package SuperExactTest (35), with the total gene number in the genome being set as 30,000.

### Cross-Trait Meta-Analysis

Given that MDD has the closest relationship with stroke among the CVD, we performed a cross-trait meta-analysis of the MDD and the stroke using the subset-based fixed-effects method ASSET (version 2.4.0) (36). The meta-analysis pools the effect of a given SNP across K studies, weighting the effects by the size of the study under the default parameters. After subset-based meta-analysis, SNPs with *P*-values lower than 5 × 10^−8^ were considered statistically significant. FUMA was used for functional annotation and gene-mapping of variants and identify LD-independent genomic regions in the meta-analysis result (37). Enrichment of the shared genes in the GWAS catalog reported categories was calculated using FUMA (37). Gene property analysis for tissue specificity was performed by FUMA. To ensure that sample overlap did not contribute to inflated estimates of genetic overlap between MDD and stroke, λmeta statistics were calculated (38). The λmeta is a statistic that uses effect size concordance to detect sample overlap or heterogeneity. Under the null hypothesis, λmeta = 1 when the pair of cohorts are completely independent. When there are overlapping samples, λmeta <1. When there is heterogeneity between datasets, the expectation is λmeta > 1. In most GWAS meta-analyses, λmeta is likely to be slightly larger than 1 due to unknown heterogeneity.

### Fine-Mapping of TWAS Associations

To prioritize putatively causal genes, we used fine-mapping of causal gene sets (FOCUS v0.6.10) (39) to the meta-analysis MDD and stroke results in three relevant tissues, including the brain, whole blood, and heart. FOCUS models predict expression correlations and assign a posterior inclusion probability (PIP) for genes at each transcriptome-wide association study (TWAS) region and relevant tissue types. A multi-tissue eQTL reference weight database was employed, and LD information from LDSC was used as reference. Multiple testing corrections were used to account for all gene–tissue pairs using Benjamini–Hochberg adjusted TWAS *P*-values (FDR < 0.05).

## Results

### Genetic Correlation and Polygenic Overlap Analysis

**Figure 1:**
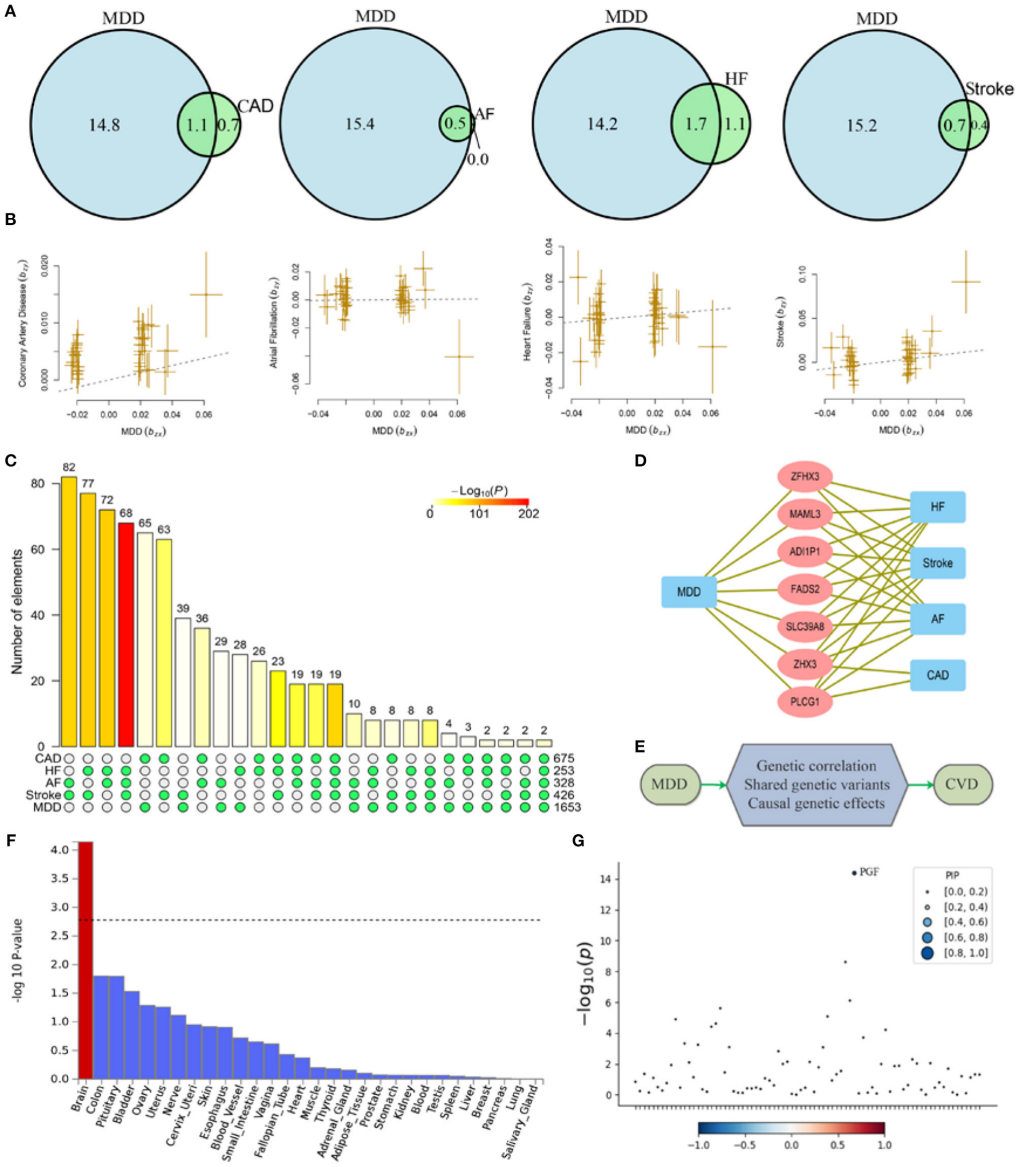
Shared causal variants and causal effects between MDD and CVD. MDD, major depressive disorder; HF, heart failure; AF, atrial fibrillation; CAD, coronary artery disease. **(A)** Venn diagrams of unique and shared causal variants between major depressive disorder and cardiovascular diseases. The numbers indicate the estimated quantity of causal variants (in thousands) per component, explaining 90% of SNP heritability in each phenotype. The size of the circles reflects the degree of polygenicity. **(B)** Causal effects of MDD on cardiovascular diseases. The dotted lines denote effect sizes (b_xy_). **(C)** Overlapped genes between major depressive disorder and cardiovascular disease from GWAS-catalog. The matrix of solid and empty circles at the bottom illustrates the “presence” (solid green) or “absence” (empty) of the gene sets in each intersection. The numbers to the right of the matrix are set sizes. The colored bars on the top of the matrix represent the intersection sizes with the color intensity showing the *P*-value significance. **(D)** Pleiotropic genes shared by MDD and CVD. **(E)** Mechanisms underlying associations between MDD and CVD. **(F)** Gene property analysis for tissue specificity in general GTEx tissues. **(G)** Fine-mapping of TWAS hits within 14:72890537-14:76444767 in heart_left_ventricle. Transcriptome-wide association signal indicating the strength of predicted expression associated with the trait.

Genetic correlation analyses indicated that MDD has a significant genetic correlation with CAD, heart failure, atrial fibrillation, and pulse pressure (Table 2). Polygenic overlap analysis indicated that 15.8 thousand variants causally influence MDD, while CVD was associated with much smaller numbers of causal variants, ranging from 0.5 thousand for the atrial fibrillation to 2.8 thousand for heart failure. Each of the tested CVD or cardiovascular measurements has shared a substantial set of causal variants with that of MDD (Figure 1A).

**Table 2:** Genetic correlation and Mendelian randomization analysis.

**Trait**	**Genetic correlation**			**Mendelian randomization**				**Reverse mendelian randomization**		
	**r_**g**_ (s.e.)**	***P***	**FDR**	**b_**xy**_ (s.e.)**	***P***	**FDR**	***N***	**b_**xy**_ (s.e.)**	***P***	***N***
Coronary artery disease	0.157 (0.027)	3.85 × 10^−9^	1.35 × 10^−8^	0.063 (0.020)	1.92 × 10^−3^	6.72 × 10^−3^	38	−0.002 (0.011)	0.882	72
Heart failure	0.227 (0.036)	4.21 × 10^−10^	2.95 × 10^−9^	0.068 (0.061)	0.268	0.375	47	0.075 (0.095)	0.432	12
Atrial fibrillation	0.067 (0.027)	0.012	0.028	0.008 (0.056)	0.88	0.882	45	0.011 (0.023)	0.631	186
Stroke	0.069 (0.042)	0.096	0.134	0.190 (0.073)	9.14 × 10^−3^	0.021	46	0.011 (0.023)	0.631	89^a^
Systolic blood pressure	−0.017 (0.017)	0.338	0.338	−0.305 (0.247)	0.217	0.375	41	−0.011 (0.016)	0.518	164
Diastolic blood pressure	0.018 (0.017)	0.266	0.31	0.058 (0.141)	0.680	0.793	42	0.003 (0.016)	0.854	149
Pulse pressure	−0.045 (0.019)	0.016	0.028	−0.556 (0.165)	7.33 × 10^−4^	5.13 × 10^−3^	43	−0.017 (0.017)	0.316	184

*s.e., standard error*;

a *P-value threshold of 1 × 10^−5^ was used. Reverse Mendelian randomization denotes causal effects of cardiovascular conditions on major depressive disorder*.

### MR Analysis

MDD confers a causal effect on CAD, stroke, and pulse pressure (Table 2, Figure 1B). Positive causal effects of MDD on stroke (b_xy_ = 0.19) were the largest among the cardiovascular conditions profiled. Of note, causal effects of MDD on pulse pressure were negative (b_xy_ = −0.56), indicating that liability to MDD may result in decreased pulse pressure. However, in general, we show that cardiovascular conditions do not confer a causal effect on MDD.

### Validation of Genetic Correlation and MR Analysis

In the validation stage, we examined the genetic correlation and causal associations between MDD and CVD. Our results indicate that MDD has a significant genetic correlation with CVD (r_g_ = 0.357, s.e. = 0.056, *P* = 1.79 × 10^−10^). Genetic liability to MDD confers a causal effect on CVD (b_xy_ = 0.26, s.e. = 0.10, *P* = 9.84 × 10^−3^), while genetic liability to CVD confers a causal effect on MDD (b_xy_ = 0.07, s.e. = 0.03, *P* = 4.74 × 10^−3^). However, the causal effect conferred by CVD on MDD was relatively weak.

### Overlapped Genes Between MDD and CVD

There were 675, 253, 328, 426, and 1,653 genome-wide significant genes for CAD, heart failure, atrial fibrillation, stroke, and MDD, respectively. There was an over-representation of shared genes between MDD and each of the four types of CVD (Figure 1C, Supplementary Table 1). A total of seven pleiotropic genes were implicated in MDD and at least three types of CVD, including *SLC39A8, MAML3, FADS2, ZFHX3, PLCG1, ZHX3*, and *ADI1P1* (Figure 1D). Notably, *ZHX3* and *ADI1P1* genes were shared by MDD with all four types of CVD.

### Cross-Trait Meta-Analysis

**Figure 2:**
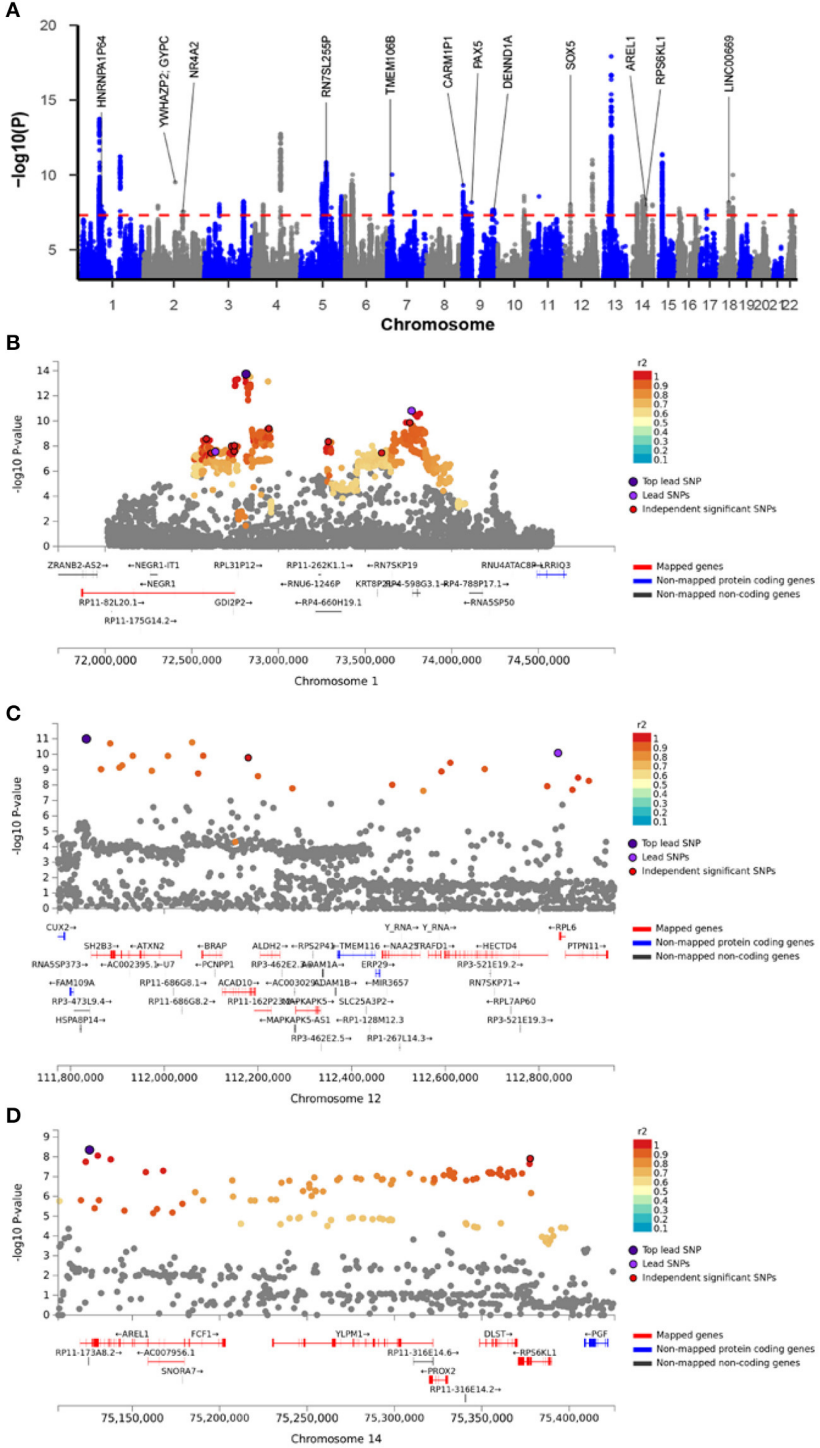
Meta-analysis of major depressive disorder with stroke. **(A)** Manhattan plot of the meta-analysis. The x-axis is the chromosomal position of SNPs and the y-axis is the significance of the SNPs (-log_10_P). Genes implicated by independent significant SNPs were annotated. **(B–D)** The four highlighted genomic loci. Each SNP is color-coded based on the highest *r*^2^ to one of the independent significant SNPs if that is greater or equal to the *r*^2^ threshold of 0.6. Other SNPs (below the *r*^2^ of 0.6) are colored in gray.

The cross-trait meta-analysis of MDD and stroke revealed 45 loci with 104 independent significant SNPs (IndSigSNPs), including 13 loci involving 19 pleiotropic IndSigSNPs and associated with both traits (Table 3, Figures 2A–D). Tissue expression analysis showed that the associations were significantly enriched in brain tissues (Figure 1F). For datasets on MDD and stroke, λmeta values were at 1.11 ± 0.01, indicating no significant overlap between disease-specific GWAS samples. Quantile-quantile (QQ) plots to display the observed meta-analysis statistics vs. the expected statistics under the null model of no associations in the -log_10_(p) scale are shown in Supplementary Figure 1.

**Table 3:** Genomic loci shared between major depressive disorder and stroke.

**No**	**Lead SNP**	**Chr**	**BP**	**Start:End**	***P***	**Genes**
1	rs61453857	1	80893110	80784642:80900786	1.23 × 10^−8^	HNRNPA1P64
2	rs1568452	2	58012833	57942325:58237405	1.17 × 10^−8^	VRK2
3	rs76485002	2	127342267	127342267:127342267	3.07 × 10^−10^	YWHAZP2, GYPC
4	rs12994955	2	157116975	157014004:157150188	2.96 × 10^−8^	NR4A2
5	rs73102900	3	61337306	61337306:61355422	9.08 × 10^−9^	
6	rs12658032	5	103904226	103671867:104082179	1.46 × 10^−11^	RN7SL255P
7	rs4721058	7	12267256	12233848:12285140	1.99 × 10^−9^	TMEM106B, VWDE
8	rs4741790	9	2977388	2935580:2998222	4.96 × 10^−10^	CARM1P1
9	rs3824344	9	37000687	36999369:37001471	6.86 × 10^−9^	PAX5
10	rs7029033	9	126682068	126573102:126688136	2.18 × 10^−8^	DENND1A, PIGFP2
11	rs7968921	12	23960177	23929026:23979791	9.15 × 10^−9^	SOX5
12	rs7152906	14	75125540	75108290:75397764	4.45 × 10^−9^	AREL1, FCF1, YLPM1, PROX2, DLST, RPS6KL1, PGF
13	rs2163544	18	36885075	36777092:36904968	6.58 × 10^−9^	LINC00669

*Chr, chromosome; BP, base position*.

### Fine-Mapping of TWAS Associations

To prioritize putatively causal genes from the meta-analysis of MDD and stroke, fine-mapping of TWAS associations was performed. A total of 100 gene-tissue pairs were identified as part of the 90% credible set for the three tissues, with 71 genes in total. Four genes were identified to be in the credible set with the highest posterior probabilities (PIP > 0.90), including *RPL31P12, BORCS7, PTPN11*, and *PGF* (Supplementary Table 2, Figure 1G, Supplementary Figures 2–5).

## Discussion

Depression is a major cause of morbidity and poor quality of life among CVD patients (6), and an independent risk factor for major adverse cardiovascular events (8). The comorbidity of depression and adverse cardiovascular outcomes typically forms a vicious cycle, known to significantly impact both the course and the management of these common conditions.

The polygenicity of MDD is much higher than that of CVD. Although the genetic correlation between MDD and CVD is relatively low, the substantial polygenic overlap between MDD and CVD was evident. For each CVD or related physiological parameter, more than 60% of genetic variants overlap with those of MDD. Notably, nearly all causal variants influencing atrial fibrillation risk also affect MDD. In addition, we observed an over-representation of shared genes between MDD and all types of CVD. Interestingly, two genes locating at chromosome 20q12, *PLCG1*, and *ZHX3*, were implicated in all the five traits, making the chromosome 20q12 region a major pleiotropic locus for both MDD and CVD.

The gene *PLCG1* encodes protein PLCγ1, which plays a key role in the intracellular transduction of the signal from receptor-mediated tyrosine kinase activators. In the brain, PLCγ is primarily activated by neurotransmitters, neurotrophic factors, and hormones. Prior studies have reported the potential role of *PLCG1* in both normal brain function and brain disorders, including MDD (40, 41). On the other hand, the PLCγ1-dependent signaling is critical for arterial development (42), the repair of the intima after vessel injury (43), and the myogenic constriction of cerebral arteries (44). The *ZHX3* gene encodes a member of the zinc fingers and homeoboxes (ZHX) gene family. Dysregulation of ZHX factors has been reported in both neurological and hematological diseases (45).

Even as high comorbidity of MDD and CVD has long been acknowledged, and their associations have been well-studied and discussed (7, 11), causal relationships between these two conditions came into the focus just recently. In this work, genetic liability to MDD was shown to etiologically influence the development of CAD and stroke, while liability to cardiovascular outcomes exerted no or minimal influence on MDD. Genetic correlation evaluates the relationship between two traits, and the sign of the correlation coefficient is determined by whether the directions of the shared genetic effect are predominantly the same or opposite for the two traits. Two traits can have substantial polygenic overlap with a non-significant genetic correlation between them (10, 46), which may account for the causal effect of MDD on stroke in the context of no genetic correlation between them. This leads us to the argument that the high rate of cardiovascular events in MDD patients may, at least partially, follow genetic variations inherited by the patients. When compounded with an unhealthy lifestyle, including an overall reduction of the physical activity commonly seen in depressed patients, this pre-existing liability may lead to the acquisition of cardiovascular disease. On the contrary, the high rate of depression seen in CVD patients may largely be due to a psychological and physical reaction that occurs after cardiovascular events, rather than from inherited genetic liability to MDD.

A recent study by Tang et al. reported a causal association of MDD with CAD (23). As the present study was conducted in a CAD dataset which was almost twice larger than that utilized by Tang et al. (332,477 vs. 184,305 patients) and as analytic frameworks were different, our study may be interpreted as a piece of corroborating evidence for the causal effect of MDD on CAD. Another recent work reported that genetic risk factors for MDD may pleiotropically increase CAD risk in females (47). However, the causal effect of MDD on CAD uncovered in our study was relatively weak (b_xy_ = 0.06) when compared with the effects of MDD on stroke (b_xy_ = 0.19). Moreover, our results do not support a causal role of genetic liability to MDD in the development of hypertension but suggest that liability to MDD may result in a marked reduction of pulse pressure instead (b_xy_ = −0.56).

Importantly, our results point to a causal effect of MDD on stroke, thus, extending findings from Cai et al.'s study that have reported the causal effect of MDD on an increased risk of small vessel stroke, but not on a stroke of large arteries (21). The high comorbidity between MDD and stroke has long been observed, with post-stroke depression constituting a common mental health issue (48, 49). However, biological mechanisms underlying the phenotypic relationships between MDD and stroke remain largely elusive. Our meta-analysis of MDD and stroke identified 16 protein-coding genes as shared by the two traits. Among these genes, nine have been previously implicated in GWASs of depression, namely, *AREL1, DENND1A, NR4A2, PAX5, RPS6KL1, SOX5, TMEM106B, VRK2*, and *YLPM1*; none of these genes have been identified in any GWAS for stroke. Five genes have been described as genome-wide associated with cardiovascular traits, including *PGF, PROX2, DLST, TMEM106B*, and *VWDE*. Notably, *TMEM106B* was repeatedly identified as a risk gene for frontotemporal lobar degeneration (50–52). Evidence for the involvement of *TMEM106B* in depression is also compelling (20, 53, 54). Incidentally, one recent study reported *TMEM106B* as a genome-wide risk gene for CAD (55).

To identify potentially causal genes involved in MDD and stroke, we used the fine-mapping of TWAS hits implemented in FOCUS. In course of estimating the causality in three relevant tissues, a total of 71 genes were included in the 90%-credible set, including four genes with high PIP. Specifically, the genomic region 1p31.1 (Figure 2B) containing *RPL31P12* was included in the 90%-credible gene set with a posterior probability of 1.00 in the brain cerebellum. It was reported that the SNP rs10789336 in the *NEGR1* gene is associated with the expression level of *RPL31P12* in brain tissues, and also confers the risk for MDD (56). In the 10q24.32 region, *BORCS7*, a genome-wide risk gene for schizophrenia (57, 58), blood pressure (59, 60), body mass index (61), and CAD (55), had the highest PIP of 0.97 in the dorsolateral prefrontal cortex. Notably, in a PET imaging study, a SNP in this gene was associated with the altered dopaminergic function (62). Given that both stroke and MDD affect the brain, both *RPL31P12* and *BORCS7* loci are attractive as candidates conferring genetic liability for both diseases.

In the 12q24.13 region (Figure 2C), *PTPN11* entered in the credible gene set with a PIP of 0.92 for the left ventricle of the heart. Previous GWASs have implicated *PTPN11* in peripheral artery disease (63), blood pressure (64, 65), and multiple sclerosis (66). Locus *PTPN11* encodes SHP2, a member of the protein tyrosine phosphatase family that regulates a wide variety of cellular functions including cell growth, differentiation, mitotic cycle, and oncogenic transformation. In particular, SHP2 serves as a pivotal regulator of normal cardiac development and function (67). *PTPN11* mutations are the most common cause of Noonan syndrome, a relatively common autosomal dominant disorder, classified as a RASopathy (68), a disorder of RAS signaling commonly associated with hypertrophic cardiomyopathy, or other malformations of the blood vessels. Our study provides evidence supporting the potential causal role of *PTPN11* in stroke.

In the genomic locus 14q24.3 (Figure 2D), *PGF* had a PIP of 0.96 for the left ventricle of the heart. *PGF* encodes a secreted placental growth factor (PGF), which belongs to the vascular endothelial growth factor (VEGF) superfamily. PGF regulates cardiac adaptation through the hypertrophy of the heart tissue by inducing capillary growth and fibroblast proliferation (69). In the heart, PGF serves as a protective paracrine effector (70). One animal study demonstrated that the deficiency of Pgf in rodents affects cognitive functions, brain neuroanatomy, and cerebrovasculature (71). In human patients, reduced expression of PGF was linked to preeclampsia and cerebrovascular and neurological aberrations occurring in fetuses; in turn, preeclampsia may impair cognitive functioning, increase the risk for stroke and lead to adverse stroke outcomes (72). Previous genome-wide analyses identified *PGF* as a candidate gene both for CAD (55) and for mood instability (73). Our meta-analysis identified *PGF* as a risk gene for both MDD and stroke, and fine-mapping of TWAS signals further asserted that *PGF* is a possible causal gene for stroke.

In 2008, the American Heart Association (AHA) issued an advisory to screen all patients with CAD for depression (74). Later it was demonstrated that, in this group of patients, a standardized screening pathway for the assessment of depression offers the potential for early identification and improved management (75, 76). Similarly, recognition of shared genetic liability between MDD and CVD suggests the need to evaluate cardiovascular risk in patients with MDD, for example, by using polygenic risk scores (PRS). Since medical comorbidities are also known to contribute to either poor response to antidepressants or treatment resistance (77), it is tempting to speculate that a stratified allocation of treatment for MDD patients with higher genetic risk for CVD may help both to achieve a better response to SSRIs and to lower the risk for an adverse outcome of CVD.

Together, our study reveals novel mechanisms by which MDD influences the risk for the development of CVD (Figure 1E). Identification of shared genetic foundations for MDD and CVD may guide drug discovery and inform early prediction and personalized treatment for these commonly comorbid conditions.

The presented study has several strengths. First, to evaluate the shared genetic liability between MDD and CVD multiple cardiovascular outcomes were analyzed. Second, for each trait, we typically prioritized the largest available dataset as a study backbone. Furthermore, to avoid potential population heterogeneity across the studies, whenever possible, we limited our analysis to individuals of European ancestry. Finally, the genetic relationships between MDD and CVD were evaluated using multiple analytic strategies, corroborating each other.

We should acknowledge several limitations of this work. As our analyses were limited to a genetic component of the traits and European ancestry population, the presented results should be interpreted cautiously. It is also worth noticing that TWAS associations are not free of noise, since the gene expression levels were imputed from weighted linear combinations of SNPs. Considering that the observed causal effect of MDD on CAD was relatively weak, only stroke was included in the further gene-hunting analyses, thus, minimizing the possibility of overreaching for causal inference.

## Conclusion

MDD and major types of CVD share substantial genetic variations. Genetic liability to MDD may confer risk for stroke and CAD. Presented results shed light on mechanisms underlying phenotypic relationships between MDD, CVD, and prioritize several candidate genes for future studies.

## Data Availability Statement

All datasets analyzed during this study are publicly available at PGC (https://www.med.unc.edu/pgc/) and GWAS catalog (https://www.ebi.ac.uk/gwas/).

## Author Contributions

FZ conceived the project and analyzed the data. FZ and AB wrote the manuscript. FZ, AB, and HC contributed to the revision of the manuscript. All authors read and approved the final manuscript.

## Funding

This work was supported by the National Natural Science Foundation of China (81471364).

## Conflict of Interest

The authors declare that the research was conducted in the absence of any commercial or financial relationships that could be construed as a potential conflict of interest.

## Publisher's Note

All claims expressed in this article are solely those of the authors and do not necessarily represent those of their affiliated organizations, or those of the publisher, the editors and the reviewers. Any product that may be evaluated in this article, or claim that may be made by its manufacturer, is not guaranteed or endorsed by the publisher.
