# Supplementary material for: Shared Genetic Liability and Causal Associations Between Major Depressive Disorder and Cardiovascular Diseases
Source: Front Cardiovasc Med. 2021 Nov 11;8:735136. doi: 10.3389/fcvm.2021.735136 (PMC8631916; doi:10.3389/fcvm.2021.735136)
Supplement: Supplementary file 1 [file Data_Sheet_1.doc]

**Supplementary File**

1. Methods

1.1 Dataset description and subject details

1.2 LD score regression

1.3 Polygenic overlap analysis

1.4 Cross-trait meta-analysis

1.5 MR analysis

1.6 Fine-mapping of TWAS associations

1.7 Quality control of GWAS datasets

2. Supplementary tables

2.1 Supplementary Table 1. Pleiotropic genes across major depressive disorder and cardiovascular diseases

2.2 Supplementary Table 2. Fine-mapping of transcriptome-wide association signals

3. Supplementary figures

3.1 Supplementary Figure 1. Quantile-quantile plots of the observed meta-analysis statistics versus the expected statistics for major depressive disorder and stroke.

3.2 Supplementary Figure 2. Fine-mapping of TWAS hits within 1:71684831-1:74326484 in brain_cerebellum.

3.3 Supplementary Figure 3. Fine-mapping of TWAS hits within 10:104380686-10:106695048 in brain_dorsolateral_prefrontal_cortex.

3.4 Supplementary Figure 4. Fine-mapping of TWAS hits within 12:110336719-12:113263518 in heart_left_ventricle.

3.5 Supplementary Figure 5. Fine-mapping of TWAS hits within 14:72890537-14:76444767 in heart_left_ventricle.

4. References

**1. Methods**

**1.1 Dataset description and subject details**

**Major depressive disorder | Wray et al., 2018**

The major depressive disorder (MDD) dataset includes 135,458 cases and 344,901 controls from seven case-control cohorts (1). A total of 44 loci were identified as associated with major depression. The first cohort included 29 case-control samples of European descent where lifetime diagnosis of major depressive disorder was ascertained using structured clinical interviews (DSM-V, ICD-9, or ICD-10), clinician-administered checklists, or review of medical records. Six additional cohorts of European ancestry, including the Hyde et al. study (23andMe Inc.), determined case status using other methods including national or hospital treatment registers, self-reported symptoms or treatment by a medical professional, or direct interviews.

**Coronary artery disease | Nelson CP, et al., 2017**

The dataset was a meta-analysis of UK Biobank SOFT CAD GWAS (interim release) with CARDIoGRAMplusC4D 1000 Genomes-based GWAS (dataset 4) and the Myocardial Infarction Genetics and CARDIoGRAM Exome (dataset 5) (2). The SOFT CAD phenotype encompasses individuals with fatal or nonfatal myocardial infarction (MI), percutaneous transluminal coronary angioplasty (PTCA) or coronary artery bypass grafting (CABG), chronic ischemic heart disease (IHD) and angina. The discovery sample comprised 71,602 cases and 260,875 controls.

**Heart failure | Shah S, et al., 2020**

Participants of European ancestry from 26 cohorts (with a total of 29 distinct datasets) with either a case-control or population-based study design were included in the meta-analysis, as part of the HERMES Consortium (3). Cases included participants with a clinical diagnosis of heart failure (HF) of any etiology with no inclusion criteria based on LV ejection fraction; controls were participants without HF. The authors meta-analyzed data from a total of 47,309 cases and 930,014 controls. The meta-analysis results have been filtered for SNPs that were not present in more than 50% of studies.

**Atrial fibrillation | Roselli C, et al., 2018**

Participants were collected from both case-control studies for atrial fibrillation (AF) and population-based studies (4). Participants from more than 50 studies were included in this analysis. The majority of studies were part of the Atrial Fibrillation Genetics (AFGen) consortium and the Broad AF study (Broad AF). Additional summary-level results from the UK Biobank (UKBB) and Biobank Japan (BBJ) were included. Cases include participants with paroxysmal or permanent AF or atrial flutter, and referents were free of these diagnoses. Ascertainment of AF in the UKBB includes samples with one or more of the following codes: non-cancer illness code, self-reported (1471, 1483); operation code (1524); diagnoses - main/secondary ICD10 (I48, I48.0-4, I48.9); underlying (primary/secondary) cause of death: ICD10 (I48, I48.0-4, I48.9); diagnoses - main/secondary ICD9 (4273); operative procedures - main/secondary OPCS (K57.1, K62.1-4). The authors analyzed 55,114 cases and 482,295 referents of European ancestry, 1,307 cases and 7,660 referents of African American ancestry, 8,180 cases and 28,612 referents of Japanese ancestry, 568 cases and 1,096 referents from Brazil, and 277 cases and 3,081 referents of Hispanic ethnicity. Samples from the UKBB, the Broad AF study and some studies from the AFGen consortium (SiGN, EGCUT, PHB and the Vanderbilt Atrial Fibrillation Registry) were previously not included in primary AF GWAS discovery analyses. There is minimal sample overlap from the studies MGH AF, BBJ and AFLMU between this and previous analyses. The atrial fibrillation dataset includes 84.2% European participants and 15.7% non- European participants.

**Stroke | Malik R, et al., 2018**

The stroke GWAS dataset was a fixed-effects meta-analysis restricted to Europeans (40,585 cases; 406,111 controls) (5). Results for any stroke was used in the analysis.

**Systolic blood pressure, Diastolic blood pressure, and Pulse pressure | Evangelou E, et al., 2018**

The dataset came from the discovery meta-analysis of both UKB and ICBP (N~750k), for all ~7 million post-QC SNPs imputed from the HRC reference panel, which were common to both the UKB and ICBP datasets (6). All participants were of European ancestry.

The UKB data was from a GWAS analysis of 458,577 UKB participants. These consist of 408,951 individuals from UKB genotyped at 825,927 variants with a custom Affymetrix UK Biobank Axiom Array chip and 49,626 individuals genotyped at 807,411 variants with a custom Affymetrix UK BiLEVE Axiom Array chip from the UK BiLEVE study, which is a subset of UKB. SNPs were imputed centrally by UKB using a reference panel that merged the UK10K and 1000 Genomes Phase 3 panel as well as the Haplotype Reference Consortium (HRC) panel. Only SNPs imputed from the HRC panel were considered.

International Consortium for Blood Pressure (ICBP) GWAS is an international consortium to investigate blood pressure genetics. The authors combined previously reported post-QC GWAS data from 54 studies (n=150,134), with newly available GWAS data from a further 23 independent studies (n=148,890) using a fixed-effects inverse-variance-weighted meta-analysis. The 23 studies providing new data were ASCOT-SC, ASCOT-UK, BRIGHT, Dijon 3C, EPICCVD, GAPP, HCS, GS:SFHS, Lifelines, JUPITER, PREVEND, TWINSUK, GWASFenland, InterAct-GWAS, OMICS-EPIC, OMICS-Fenland, UKHLS, GoDARTSIllumina and GoDarts-Affymetrix, NEO, MDC, SardiNIA and METSIM. All study participants were Europeans and were imputed to either the 1000 Genomes Project Phase 1 integrated release v.3 (March 2012) all-ancestry reference panel or the HRC panel. The final enlarged ICBP GWAS dataset included 77 cohorts (n=299,024).

**Cardiovascular disease | Sudlow, 2015**

The cardiovascular disease (CVD) included angina, heart attack/myocardial infarction, heart/cardiac problem, irregular heart beat, heart failure/pulmonary odema, atrial fibrillation, atrial flutter, wolff parkinson white/wpw syndrome, sick sinus syndrome, svt/supraventricular tachycardia, hypertrophic cardiomyopathy (hcm/hocm), myocarditis, rheumatic fever, subarachnoid haemorrhage, brain haemorrhage, transient ischaemic attack (tia), subdural haemorrhage/haematoma, cerebral aneurysm, aortic dissection, venous thromboembolic disease, deep venous thrombosis (dvt), cardiomyopathy, aortic aneurysm, aortic aneurysm rupture, ischaemic stroke, heart arrhythmia, cerebrovascular disease.

**1.2 LD score regression**

LD score regression software v1.0.1 was used to analyze the genetic correlation of MDD with cardiovascular diseases (CVDs). The 1000 Genome project phase 3 (7) were used to estimate the LD structure for European populations, which was obtained from the LD score regression website (8-10). SNPs were filtered by 1.1 million variants, a subset of 1000 Genomes and HapMap3 (11), with MAF above 0.05, MHC and other long-range LD regions excluded.

**1.3 Polygenic overlap analysis**

Frei et al. introduced a novel statistical framework (MiXeR) to quantify polygenic overlap irrespective of genetic correlation between traits (12). In MiXeR, a causal mixture model (12, 13) is used to estimate the percentage of variance explained by genome-wide significant SNPs as a function of sample size. For each SNP, i, MiXeR models its additive genetic effect of allele substitution, β_i_, as a point-normal mixture,
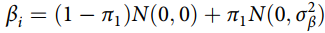
, where π_1_ represents the proportion of non-null SNPs (polygenicity) and σ^2^_β_ represents the variance of effect sizes of non-null SNPs. Then, for each SNP, j, MiXeR incorporates LD information and allele frequencies for 9,997,231 SNPs extracted from 1000 Genomes Phase 3 data (8-10) to estimate the expected probability distribution of the signed test statistic,
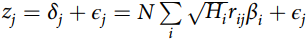
, where N is the sample size, H_i_ indicates heterozygosity of ith SNP, r_ij_ indicates the allelic correlation between the *i*th and *j*th SNPs, and ϵ_j_ ~ N(0, σ^2^_0_) is the residual variance. Further, the three parameters, π_1_, σ^2^_β_, and σ^2^_0_, are fitted by direct maximization of the likelihood function. In the MiXeR analysis, an effective sample size of Neff = 4/(1/Ncase + 1/Ncontrol) was used to account for imbalanced numbers of cases and controls. The python_convert (v0.9.2) pipeline was used to harmonize GWAS summary statistics (https://github.com/precimed/python_convert).

**1.4 Cross-trait meta-analysis**

ASSET meta-analysis is an agnostic approach that generalizes standard fixed-effects meta-analysis by allowing a subset of the input GWASs to have no effect on a given SNP. The method exhaustively explores all possible subsets of ‘‘non-null’’ GWAS inputs within a fixed-effect framework to identify the strongest association signal in both positive and negative directions. Only SNPs that were present for both the traits were retained as inputs to the ASSET meta-analysis, resulting in 8,243,736 SNPs for subsequent analysis. We carried out a one-sided analysis by exploring models in which all non-null studies have effects in the same direction. Default parameters were applied with the ‘‘h.traits’’ function in ASSET.

FUMA was used to map SNPs to genes and identify LD-independent genomic regions (14). The nearest genes and functional consequence of each SNP on gene functions were annotated based on ANNOVAR (15). Firstly, independent signiﬁcant SNPs (IndSigSNPs) were identiﬁed based on their *P*-value being genome-wide signiﬁcant (*P* ≤ 5.0×10^−8^) and being independent of each other (r^2^ < 0.6). Secondly, Lead SNPs were identiﬁed as a subset of the independent signiﬁcant SNPs that were in LD with each other at r^2^ < 0.1 within a 1 Mb window. Genomic risk loci were identified by merging lead SNPs if they were closer than 500 kb apart. Clumping procedures were carried out on the basis of the European 1000 Genomes Project phase 3 reference panel. Due to extensive LD, the MHC region was merged into one region (chr6:25-35Mb). Genes within 100 kb of each variant were mapped.

**1.5 MR analysis**

To infer credible causal associations between MDD and CVDs, we performed Mendelian randomization analysis using GSMR V1.0.9 (16). This method utilizes summary-level data to test for putative causal associations between a risk factor (exposure) and an outcome by using independent genomewide significant SNPs as instrumental variables as an index of the exposure. HEIDI outlier detection was used to filter genetic instruments that showed clear pleiotropic effects on the exposure phenotype and the outcome phenotype. We used a threshold P-value of 0.01 for the outlier detection analysis in HEIDI, which removes 1% of SNPs by chance if there is no pleiotropic effect. We tested for bidirectional causation by repeating the analyses while switching the role of each cardiovascular phenotype as exposure and MDD as the outcome. For each trait, we selected independent (r^2^ = 0.01), genome-wide significant lead SNPs as instrumental variables in the analyses.

The method estimates a putative causal effect of the exposure on the outcome (b_xy_) as a function of the relationship between the SNP’s effects on the exposure (b_zx_) and the SNP’s effects on the outcome (b_zy_), given the assumption that the effect of non-pleiotropic SNPs on an exposure (x) should be related to their effect on the outcome (y) in an independent sample only via mediation through the phenotypic causal pathway (b_xy_). The estimated causal effect coefficients (b_xy_) are approximately equal to the natural log odds ratio for a case-control trait. An odds ratio of 2 can be interpreted as a doubled risk compared with the population prevalence of a binary trait for every standard deviation increase in the exposure trait.

**1.6 Fine-mapping of TWAS associations**

FOCUS (Fine-mapping Of CaUsal gene Sets) is software to fine-map transcriptome-wide association study (TWAS) statistics at genomic risk regions. The software takes as input summary GWAS data along with eQTL weights and outputs a credible set of genes to explain the observed genomic risk. FOCUS v0.6.10 was used to identify potentially causal genes from the meta-analysis result of MDD and stroke (17). A multiple tissues, multiple eQTL reference panel weight database from the software (https://github.com/bogdanlab/focus/) was used. This combines GTEx v7 weights from PrediXcan (18) with METSIM, NTR, YFS, and CMC weights from FUSION software (19) into a single usable database for FOCUS. By integrating the GWAS summary result, expression weights, and LD among SNPs, it identifies causal gene to be included in a 90%-credible set and give a posterior probability (PIP) to estimate the causality in relevant tissues.

We applied FOCUS analysis on the meta-analysis result of MDD and stroke in three relevant tissues, including the brain (involving 13 brain tissues), blood, and heart. The results from the three tissues were merged and irrelevant tissues other than the four tissues were removed.

**1.7 Quality control of GWAS datasets**

We compared SNP alleles between the MDD dataset and each of the CVD datasets. 1) SNPs were filtered based on INFO ≥0.80 if it exists. 2) Each SNP was compared between the two datasets and SNPs with conflicting alleles between each pair of datasets were excluded; 3) If an SNP was mapped to opposite strands in the two datasets, alleles of the SNP in the second dataset were harmonized. If the reference alleles were complementary in the two datasets, the reference allele in the second dataset was converted and the effect direction was reversed. For ambiguous SNPs (with A/T or C/G alleles), we employed allele frequency (FRQ) filtering to determine whether the alleles from the two GWASs were derived from the same reference panel or not (SNPs with FRQ difference between the two GWASs ≥ 0.20 were deemed as the mapping from opposite reference panels).

**2.1 Supplementary Table 1. Pleiotropic genes across major depressive disorder and cardiovascular diseases**

| Intersections | Degree | Observed_Overlap | Expected_Overlap | P | FDR |
| --- | --- | --- | --- | --- | --- |
| HF & CAD | 2 | 26 | 5.69 | 1.48E-10 | 2.57E-10 |
| AF & CAD | 2 | 36 | 7.38 | 5.56E-15 | 1.31E-14 |
| AF & HF | 2 | 72 | 2.766 | 7.32E-82 | 9.52E-81 |
| Stroke & CAD | 2 | 63 | 9.585 | 9.06E-33 | 2.62E-32 |
| Stroke & HF | 2 | 77 | 3.59 | 9.06E-81 | 7.85E-80 |
| Stroke & AF | 2 | 82 | 4.66 | 3.13E-78 | 2.03E-77 |
| MDD & CAD | 2 | 65 | 37.19 | 9.60E-06 | 1.28E-05 |
| MDD & HF | 2 | 28 | 13.94 | 3.70E-04 | 4.37E-04 |
| MDD & AF | 2 | 29 | 18.07 | 8.55E-03 | 8.55E-03 |
| MDD & Stroke | 2 | 39 | 23.47 | 1.40E-03 | 1.52E-03 |
| AF & HF & CAD | 3 | 19 | 0.062 | 2.20E-41 | 8.17E-41 |
| Stroke & HF & CAD | 3 | 23 | 0.081 | 3.69E-49 | 1.60E-48 |
| Stroke & AF & CAD | 3 | 19 | 0.105 | 5.62E-37 | 1.83E-36 |
| Stroke & AF & HF | 3 | 68 | 0.039 | 1.12E-202 | 2.91E-201 |
| MDD & HF & CAD | 3 | 3 | 0.314 | 4.02E-03 | 4.18E-03 |
| MDD & AF & CAD | 3 | 4 | 0.407 | 8.05E-04 | 9.10E-04 |
| MDD & AF & HF | 3 | 8 | 0.152 | 5.14E-12 | 1.03E-11 |
| MDD & Stroke & CAD | 3 | 8 | 0.528 | 8.45E-08 | 1.29E-07 |
| MDD & Stroke & HF | 3 | 8 | 0.198 | 4.08E-11 | 7.58E-11 |
| MDD & Stroke & AF | 3 | 10 | 0.257 | 2.09E-13 | 4.53E-13 |
| Stroke & AF & HF & CAD | 4 | 19 | 8.84E-04 | 1.21E-76 | 6.29E-76 |
| MDD & AF & HF & CAD | 4 | 2 | 3.43E-03 | 5.81E-06 | 8.39E-06 |
| MDD & Stroke & HF & CAD | 4 | 2 | 4.45E-03 | 9.81E-06 | 1.28E-05 |
| MDD & Stroke & AF & CAD | 4 | 2 | 5.77E-03 | 1.65E-05 | 2.04E-05 |
| MDD & Stroke & AF & HF | 4 | 8 | 2.16E-03 | 9.02E-27 | 2.35E-26 |
| MDD & Stroke & AF & HF & CAD | 5 | 2 | 4.87E-05 | 1.17E-09 | 1.90E-09 |

**2.2 Supplementary Table 2**. Fine-mapping of transcriptome-wide association signals

| **Gene** | **Tissue** | **TWAS_Z** | **PIP** | **Region** |
| --- | --- | --- | --- | --- |
| RPL31P12 | brain_cerebellum | -7.51 | 1.00 | 1:71684831-1:74326484 |
| BORCS7 | brain_dorsolateral_prefrontal_cortex | -1.77 | 0.97 | 10:104380686-10:106695048 |
| PTPN11 | heart_left_ventricle | -4.87 | 0.92 | 12:110336719-12:113263518 |
| PGF | heart_left_ventricle | -5.00 | 0.96 | 14:72890537-14:76444767 |

PIP: posterior inclusion probability; TWAS: transcriptome-wide association study.

**3.1 Supplementary Figure 1.** Quantile-quantile plots of the observed meta-analysis statistics versus the expected statistics for major depressive disorder and stroke


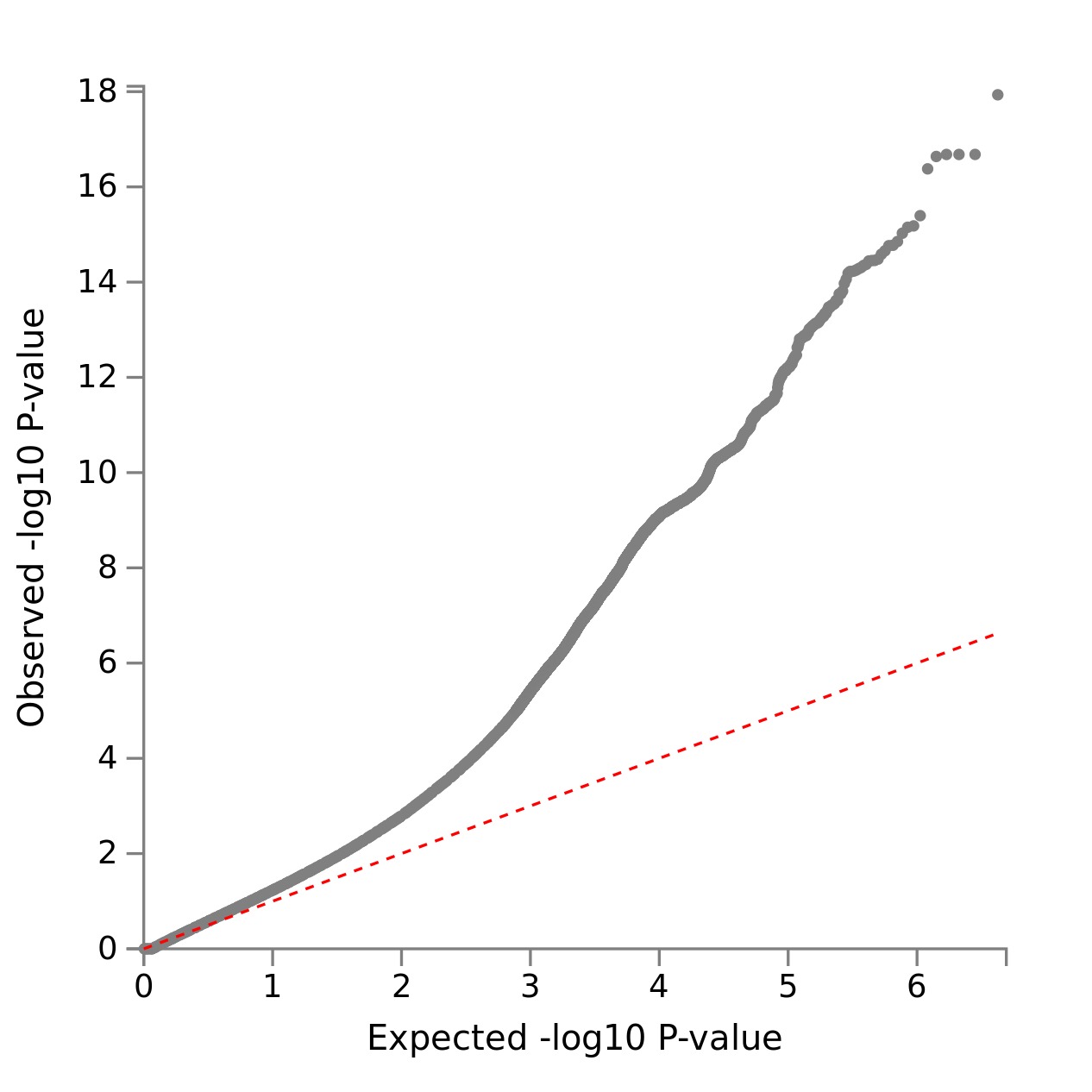


**3.2 Supplementary Figure 2. Fine-mapping of TWAS hits within 1:71684831-1:74326484 in brain_cerebellum.** Top: Transcriptome-wide association signal indicating the strength of predicted expression association with trait; Bottom: Induced correlation of predicted expression.

**
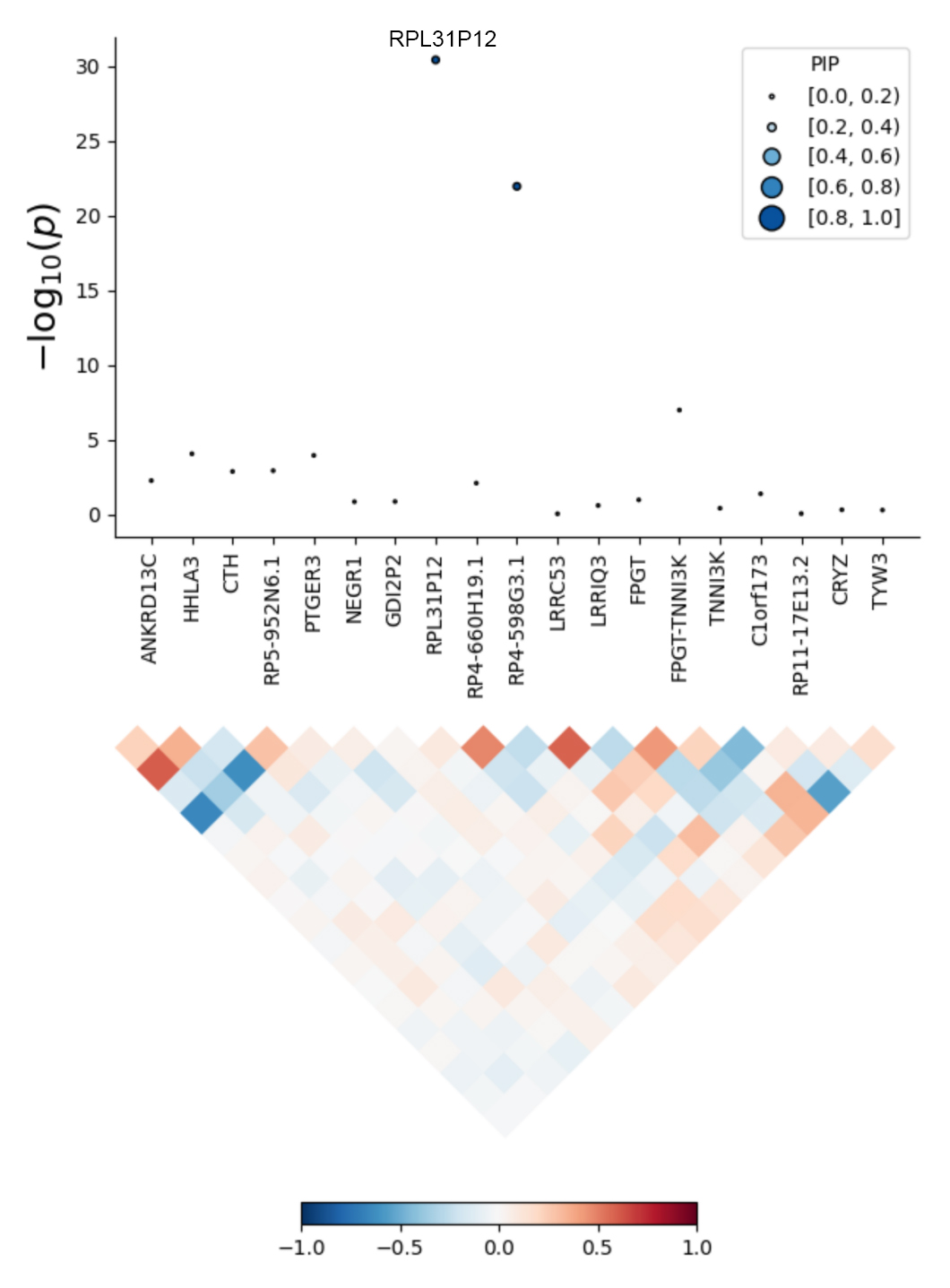
**

**3.3 Supplementary Figure 3. Fine-mapping of TWAS hits within 10:104380686-10:106695048 in brain_dorsolateral_prefrontal_cortex.** Top: Transcriptome-wide association signal indicating the strength of predicted expression association with trait; Bottom: Induced correlation of predicted expression.

**
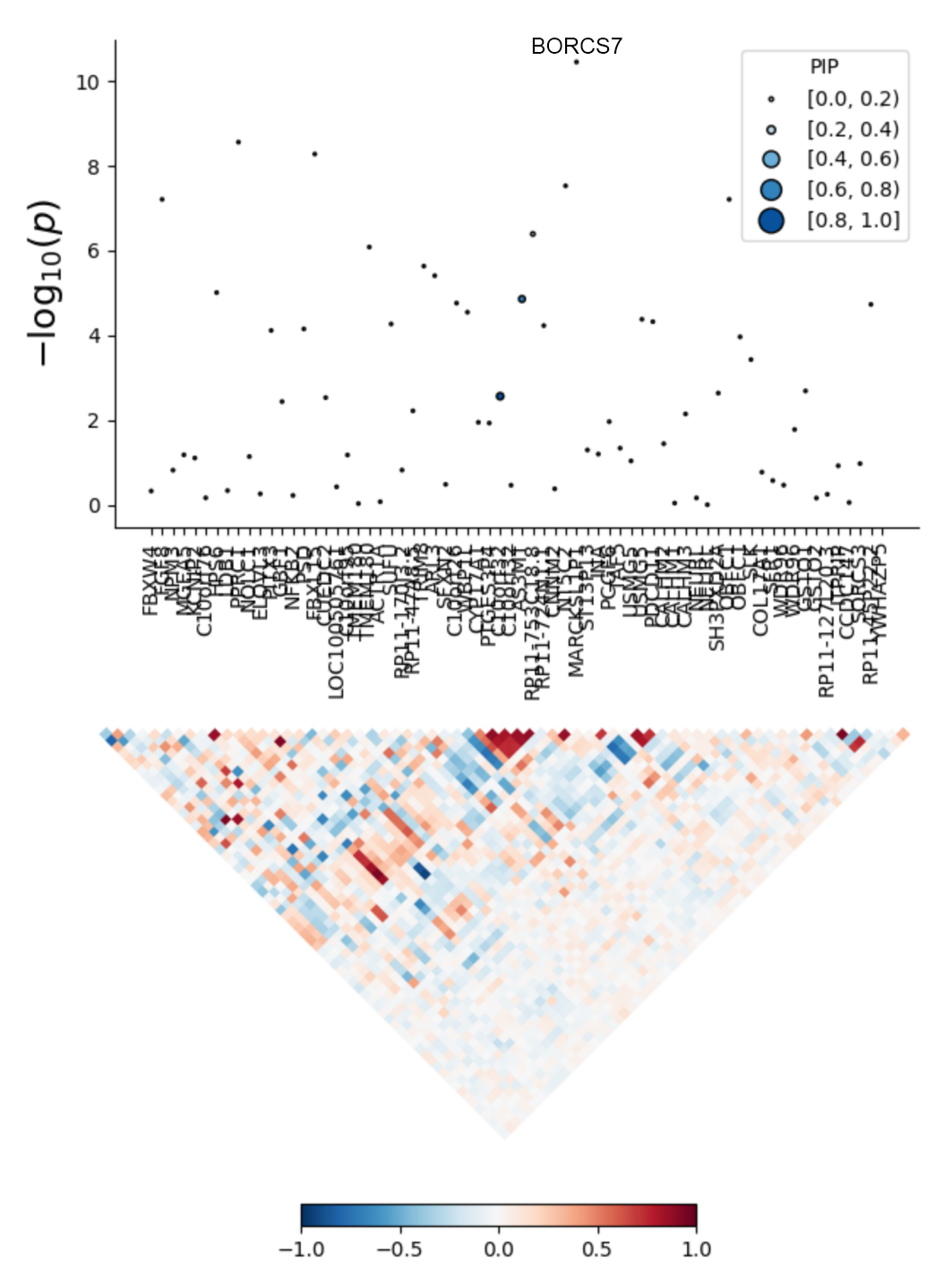
**

**3.4 Supplementary Figure 4. Fine-mapping of TWAS hits within 12:110336719-12:113263518 in heart_left_ventricle.** Top: Transcriptome-wide association signal indicating the strength of predicted expression association with trait; Bottom: Induced correlation of predicted expression.

**
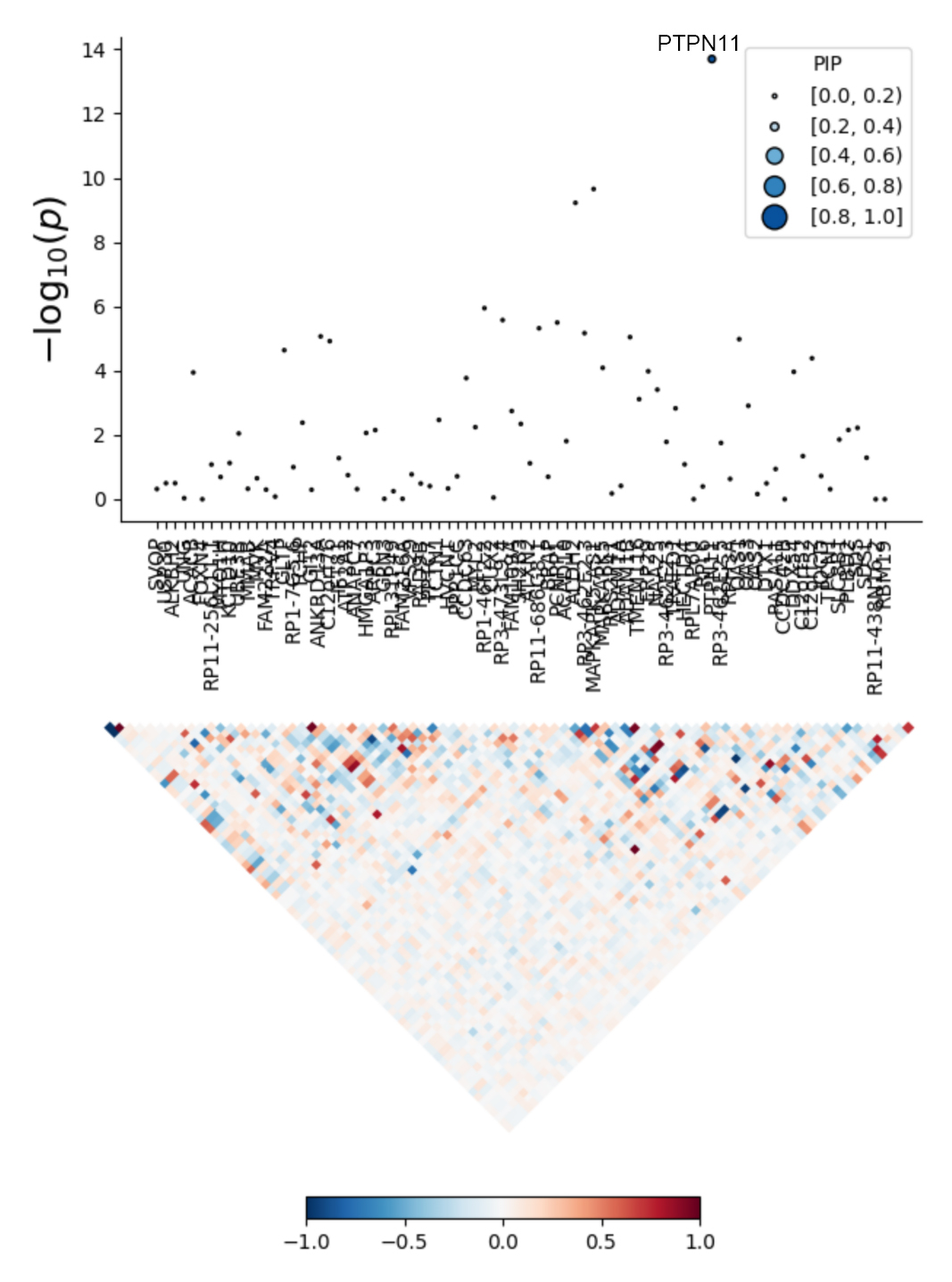
**

**3.5 Supplementary Figure 5. Fine-mapping of TWAS hits within 14:72890537-14:76444767 in heart_left_ventricle.** Top: Transcriptome-wide association signal indicating the strength of predicted expression association with trait; Bottom: Induced correlation of predicted expression.

**
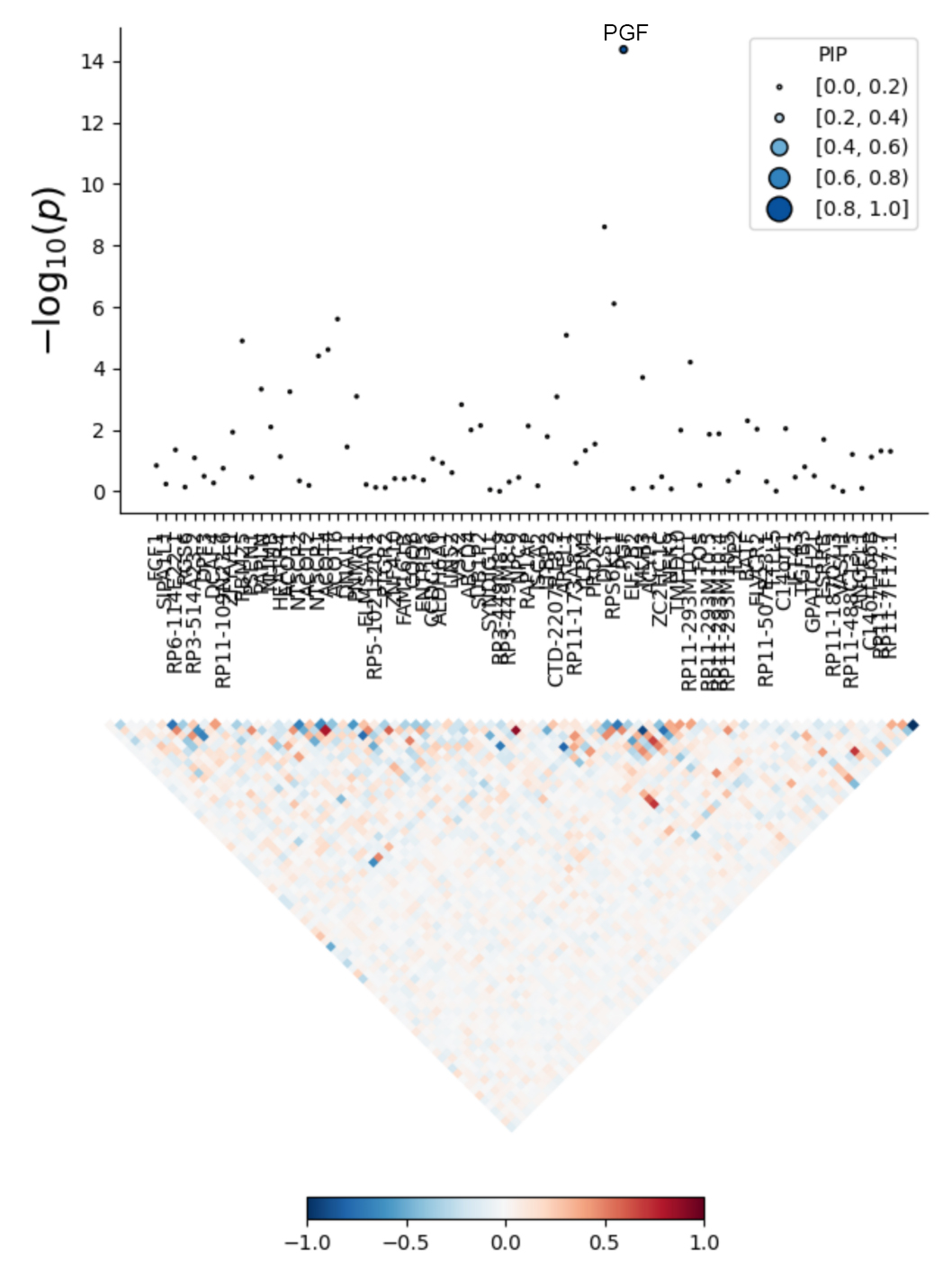
**

**4. References**

1. Wray NR, Ripke S, Mattheisen M, Trzaskowski M, Byrne EM, Abdellaoui A, et al. Genome-wide association analyses identify 44 risk variants and refine the genetic architecture of major depression. *Nat Genet* (2018) 50(5):668-681. doi: 10.1038/s41588-018-0090-3

2. Nelson CP, Goel A, Butterworth AS, Kanoni S, Webb TR, Marouli E, et al. Association analyses based on false discovery rate implicate new loci for coronary artery disease. *Nat Genet* (2017) 49(9):1385-1391. doi: 10.1038/ng.3913

3. Shah S, Henry A, Roselli C, Lin H, Sveinbjornsson G, Fatemifar G, et al. Genome-wide association and Mendelian randomisation analysis provide insights into the pathogenesis of heart failure. *Nat Commun* (2020) 11(1):163. doi: 10.1038/s41467-019-13690-5

4. Roselli C, Chaffin MD, Weng LC, Aeschbacher S, Ahlberg G, Albert CM, et al. Multi-ethnic genome-wide association study for atrial fibrillation. *Nat Genet* (2018) 50(9):1225-1233. doi: 10.1038/s41588-018-0133-9

5. Malik R, Chauhan G, Traylor M, Sargurupremraj M, Okada Y, Mishra A, et al. Multiancestry genome-wide association study of 520,000 subjects identifies 32 loci associated with stroke and stroke subtypes. *Nat Genet* (2018) 50(4):524-537. doi: 10.1038/s41588-018-0058-3

6. Evangelou E, Warren HR, Mosen-Ansorena D, Mifsud B, Pazoki R, Gao H, et al. Genetic analysis of over 1 million people identifies 535 new loci associated with blood pressure traits. *Nat Genet* (2018) 50(10):1412-1425. doi: 10.1038/s41588-018-0205-x

7. Genomes Project C, Auton A, Brooks LD, Durbin RM, Garrison EP, Kang HM, et al. A global reference for human genetic variation. *Nature* (2015) 526(7571):68-74. doi: 10.1038/nature15393

8. Bulik-Sullivan B, Finucane HK, Anttila V, Gusev A, Day FR, Loh PR, et al. An atlas of genetic correlations across human diseases and traits. *Nat Genet* (2015) 47(11):1236-1241. doi: 10.1038/ng.3406

9. Finucane HK, Bulik-Sullivan B, Gusev A, Trynka G, Reshef Y, Loh PR, et al. Partitioning heritability by functional annotation using genome-wide association summary statistics. *Nat Genet* (2015) 47(11):1228-1235. doi: 10.1038/ng.3404

10. Bulik-Sullivan BK, Loh PR, Finucane HK, Ripke S, Yang J, Schizophrenia Working Group of the Psychiatric Genomics C, et al. LD Score regression distinguishes confounding from polygenicity in genome-wide association studies. *Nat Genet* (2015) 47(3):291-295. doi: 10.1038/ng.3211

11. International HapMap C, Altshuler DM, Gibbs RA, Peltonen L, Altshuler DM, Gibbs RA, et al. Integrating common and rare genetic variation in diverse human populations. *Nature* (2010) 467(7311):52-58. doi: 10.1038/nature09298

12. Frei O, Holland D, Smeland OB, Shadrin AA, Fan CC, Maeland S, et al. Bivariate causal mixture model quantifies polygenic overlap between complex traits beyond genetic correlation. *Nat Commun* (2019) 10(1):2417. doi: 10.1038/s41467-019-10310-0

13. Holland D, Frei O, Desikan R, Fan CC, Shadrin AA, Smeland OB, et al. Beyond SNP heritability: Polygenicity and discoverability of phenotypes estimated with a univariate Gaussian mixture model. *PLoS Genet* (2020) 16(5):e1008612. doi: 10.1371/journal.pgen.1008612

14. Watanabe K, Taskesen E, van Bochoven A, Posthuma D. Functional mapping and annotation of genetic associations with FUMA. *Nat Commun* (2017) 8(1):1826. doi: 10.1038/s41467-017-01261-5

15. Wang K, Li M, Hakonarson H. ANNOVAR: functional annotation of genetic variants from high-throughput sequencing data. *Nucleic Acids Res* (2010) 38(16):e164. doi: 10.1093/nar/gkq603

16. Zhu Z, Zheng Z, Zhang F, Wu Y, Trzaskowski M, Maier R, et al. Causal associations between risk factors and common diseases inferred from GWAS summary data. *Nat Commun* (2018) 9(1):224. doi: 10.1038/s41467-017-02317-2

17. Mancuso N, Freund MK, Johnson R, Shi H, Kichaev G, Gusev A, et al. Probabilistic fine-mapping of transcriptome-wide association studies. *Nat Genet* (2019) 51(4):675-682. doi: 10.1038/s41588-019-0367-1

18. Gamazon ER, Wheeler HE, Shah KP, Mozaffari SV, Aquino-Michaels K, Carroll RJ, et al. A gene-based association method for mapping traits using reference transcriptome data. *Nat Genet* (2015) 47(9):1091-1098. doi: 10.1038/ng.3367

19. Gusev A, Ko A, Shi H, Bhatia G, Chung W, Penninx BW, et al. Integrative approaches for large-scale transcriptome-wide association studies. *Nat Genet* (2016) 48(3):245-252. doi: 10.1038/ng.3506
